# Technology Efficacy in Active Prosthetic Knees for Transfemoral Amputees: A Quantitative Evaluation

**DOI:** 10.1155/2014/297431

**Published:** 2014-07-07

**Authors:** Amr M. El-Sayed, Nur Azah Hamzaid, Noor Azuan Abu Osman

**Affiliations:** ^1^Department of Biomedical Engineering, Faculty of Engineering, University of Malaya, 50603 Kuala Lumpur, Malaysia; ^2^Mechatronics Section, Mechanical Engineering Department, Faculty of Engineering, Assiut University, Assiut 71516, Egypt

## Abstract

Several studies have presented technological ensembles of active knee systems for transfemoral prosthesis. Other studies have examined the amputees' gait performance while wearing a specific active prosthesis. This paper combined both insights, that is, a technical examination of the components used, with an evaluation of how these improved the gait of respective users. This study aims to offer a quantitative understanding of the potential enhancement derived from strategic integration of core elements in developing an effective device. The study systematically discussed the current technology in active transfemoral prosthesis with respect to its functional walking performance amongst above-knee amputee users, to evaluate the system's efficacy in producing close-to-normal user performance. The performances of its actuator, sensory system, and control technique that are incorporated in each reported system were evaluated separately and numerical comparisons were conducted based on the percentage of amputees' gait deviation from normal gait profile points. The results identified particular components that contributed closest to normal gait parameters. However, the conclusion is limitedly extendable due to the small number of studies. Thus, more clinical validation of the active prosthetic knee technology is needed to better understand the extent of contribution of each component to the most functional development.

## 1. Introduction

A prosthetic knee is the key component of a transfemoral prosthesis prescribed to above knee amputees as it replaces the functional joint. The development of prosthetic knee systems has increasingly enabled amputees to perform activities of daily living (ADL). The variation in requirements of daily behavior of the persons who lost their lower limb has encouraged scientists to continuously improve the prosthetic knee mechanisms. To understand the recent advancement of the knee technology, different knee types are classified according to its functional mechanism. The technical capability and performance of a knee prosthesis are instrumental in facilitating a walk that more closely resembles that of an able-bodied individual's biomechanics and thus increases amputee walking energy efficiency. Therefore, the extent to which the amputee can achieve normal gait biomechanics highly depends on the appropriate use and integration of sensory systems, actuators, and control scheme.

### 1.1. Passive Knee Devices

Passive knee devices have fixed impedance that is provided from either pneumatic or hydraulic systems, in which the devices' friction might change according to the walking speed of the amputees [1]. Unlike active prosthesis, passive knee types are not equipped with sensors that aid the interaction between the amputee and the environment [2, 3]. For example, mechanical-based prostheses employ passive prosthetic knee systems that have limited ability to mimic the behavior of a normal knee [4]. In a manual locking knee, a remote release cable is utilized to provide the user with stability during knee extension. However, this device leads to high energy costs during ambulation. In weight-activated knees, constant friction is used to supply high stability during stance phase. Transferring the body weight to the knee shall activate an embedded brake that prevents buckling. This brake is released once the knee is unloaded. However, constant friction is still present during the swing phase and these results are inefficient during gait. An element that is capable of storing energy, such as a spring may assist the knee during the swing phase in which it is loaded during weight bearing and released during the swing phase itself [5, 6].

Another type of passive knee device is the single axis knee, which utilizes a simple pivot mechanism. It is robust, lightweight, and relatively cheaper than other knee systems that are commonly in use. However, amputees have to use their own muscles to maintain the device stable at standing, as it is not equipped with a stance control function. Thus, users often use manual lock to compensate for lack of stance control and utilize the available friction to prevent the leg from overspeeding during the forward swing when moving into the next step [7]. Polycentric knees offer an advantage in terms of low maintenance but do not contribute towards walking pattern that resembles an able-bodied person. Other than the spring-loaded friction, polycentric knees offer little or no stance control. Stance control is a very important feature that prevents the knee from buckling in the event of an accident or an unexpected change during gait control. Nevertheless, some polycentric knees incorporate a simple locking mechanism that allows the knee to be locked in the extended position [5]. Such passive knee devices cannot adjust or control the amount of power during different terrains.

### 1.2. Powered/Motorized Knee Devices

Another type of knee prosthesis is the powered prosthetic knees that have the capability to assist amputees to perform level walking, ramp descent, stair descent, and standing and detect instances of stumble [8]. However, the devices still cannot deliver sufficient joint power to restore functions such as climbing stairs, running, many locomotive functions, and standing stability during slope terrain [9–13]. It was noted that powered knee devices can reduce the hip power demand during stance and swing phase [14]. External power delivered to the prosthetic knee enables adaptation at different walking environments. In addition, different types of sensors can be utilized to provide interaction between the prosthetic knee and the external environment. To date, there are two models of the powered knee devices: Ossur power knee and the Vanderbilt leg. Such previous models have motors that generate power to help the user to perform different ADL.

To improve the mobility of the knee prosthesis system, they incorporated the ability to restore user stability. The knee prosthesis was referred to as active device, as it is capable of delivering active response to prevent the amputee from falling due to obstacles. One example of this active powered knee can be seen in the stumble detection feature in which real-time stumble can be detected using three separate accelerometers on the prosthesis. Ten subjects were employed to validate the stumble detection through these signals and all 19 stumble responses were correctly identified [15]. Further study was performed to enhance the interaction between the user and the environment whereby new knowledge of adaptive locomotion-mode-recognition system was developed to enhance the performance of the prior system. That system was based on the integration of EMG and mechanical signals, which showed great potential at locomotion-mode recognition [16]. Electromyography-based control was successfully used to control the active knee prosthesis as presented through a set of swing experiments [17].

### 1.3. Adaptive Dissipative Knees

Adaptive dissipation knees refer to devices that can regulate the impedance by adjusting the hydraulic valves such as the Otto Bock C-Leg or the devices that use magnetorheological fluid such as Rheo knee. Microcontroller on board can regulate the impedance by tuning hydraulic valve, that is, the orifice that rectifies the flow rate. Adaptive knees adjust the resistance to control the knee after calculating the comparisons between steps and monitoring the position, velocity, forces, and moments of the prosthetic knee [14]. To differentiate between passive and active knees in terms of the advantages of each type to the transfemoral amputees, a clinical comparison was performed to identify the damping in mechanically passive and active prosthetic knee devices. The study established the idea that variable-damping knee prostheses offer transfemoral amputees significant advantages over mechanically passive designs [18]. To solve the problems associated with passive knee systems and in order to enhance amputees' functional performance, researchers started to involve electronics in the design of prosthetic knees. For example, a prosthesis that consisted of a hydraulic actuator tethered to an external source power was used to move the knee joint and subsequently control the knee [19, 20].

In general, the working principle of a smart or active device involves the integration of intrinsic computational sensors, whereby the sensors detect the physical performance of the system and this corresponds to real-time alterations in the actuator. However, an embedded controller adjusts and coordinates the knee movement accordingly [4]. The first version of an active prosthesis has been developed and the electronic prosthesis is now available as a product on the wider market [21, 22]. The Otto Bock C-Leg, Össur Rheo Knee, Adaptive2, and Synergy knee [23, 24] are all active adaptive dissipation knees. Nevertheless, the prior knee systems are still unable to generate sufficient mechanical power for normal ADLs. The experiences of nine transfemoral amputees who wore C-Leg, Rheo knee, Adaptive2, and Synergy knee were acquired in order to identify which of these commercial knee systems offer functional benefits to amputee. The results revealed that C-Leg could offer the most functional benefits during everyday gait [25].

One study compared the kinetics and kinematics performance of the C-Leg and the Mauch SNS knee prosthesis during gait [26]. The study reported that the C Leg offers low performance in terms of knee angle, knee moment, and knee power in comparison to the Mauch SNS. A review study presented transfemoral amputees' opinion of the C-Leg microprocessor-controlled prosthetic knee in terms of three categories: safety, gait energy efficiency, and cost effectiveness. The paper concluded that C-Leg is safe, energy efficient, and cost effective compared to other prosthetic knee systems [27]. Bellmann et al. (2012) produced a biomechanical comparison between the C-Leg and a new knee joint called the Genium [28]. Results of that study indicated that Genium knee facilitated more natural gait biomechanics and load distribution throughout the sound musculoskeletal structure. The biomechanical performance of amputee users who wore the Genium was compared to that of those wearing the C-Leg. The study claimed that the Genium knee joint was able to increase amputee users' activities and it has performance close to normal gait biomechanics [23].

### 1.4. Aim of Study

Despite all the commercially available and research-based transfemoral prosthesis inventions, there has not been an overall systematic analysis that directly relates the technology employed with its users' functional walking outcomes. This study was designed to establish an understanding of the existing scientific studies that addresses the development and outcome of electronic prosthetic knee for transfemoral amputees based on a biomechatronics approach [29]. In particular, the aim of this paper was to identify the available technology in terms of sensor, actuator, and control techniques that can directly benefit transfemoral amputees as they perform normal walking activities while using the available active knees, by addressing the components that were integrated in each transfemoral prosthetic design. While examining the respective benefits that have been offered to amputees and the corresponding cost-and-benefit balance, an informed decision can be made as to which components could be better integrated in future prosthetic development. In essence, there is currently a knowledge gap in terms of the performance of current prosthetic knee systems as compared to the anatomical knees, especially regarding the error between the anticipated and the real system pattern. Although significant effort has been invested in the development of knee prostheses, there are no sufficient claims about the advantages and disadvantages of the utilized sensor, actuator, or control scheme [30]. In short, the paper aims to identify the effect of the current technology involved in active knees in terms of its sensory system, actuator, and control technique to achieve normal gait performance. In particular, the authors aim to determine the degree of contribution of each component in providing the most benefits in enhancing the amputees' biomechanical walking performance. We hypothesize that every single component directly affects the amputee's gait. In the following sections, assessments of current active knee systems' components of the studies that reported the clinical trials were discussed in detail.

## 2. Methods

**Figure 1:**
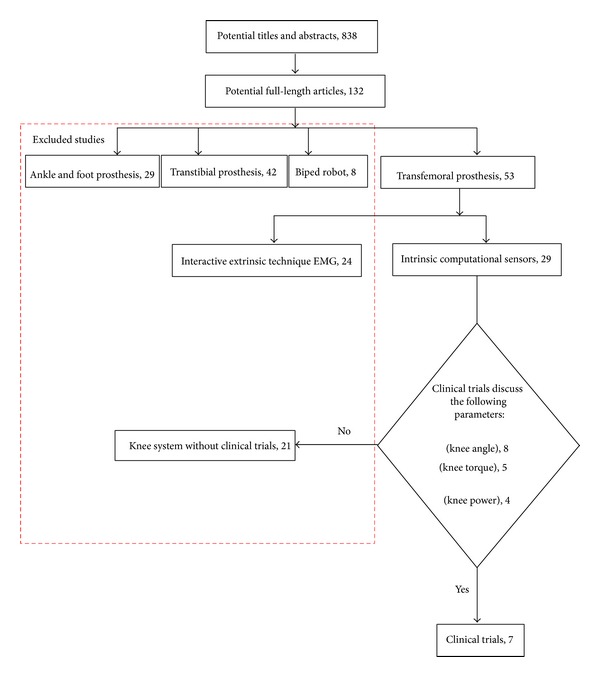
Flowchart of systematic survey of the literature based on PRISMA guideline.

Published articles were searched using different databases, namely, the Academic Source Premier at EBSCOhost (© 2012 EBSCO Industries), ACM (Association for Computing Machinery) (Copyright © 2012 ACM, Inc.), Biological Abstracts at Ovid (Copyright © 2000–2011 Ovid Technologies, Inc.), Cambridge Journals Online (Cambridge University Press 2012), Emerald (© 2012 Emerald Group Publishing Limited), IEEE Xplore (© Copyright 2012 IEEE), ScienceDirect (Copyright © 2012 Elsevier B.V), SpringerLink (© 2012 Springer, part of Springer Science + Business Media), PubMed (Copyright 2012 NCBI), and Taylor & Francis Journals (© 2012 Informa Plc). Studies were limited to English language or other languages that were translated to English. The criteria of focus were as follows: (1) sensors, (2) actuators and transmission systems, and (3) control techniques. Keywords that were used to identify suitable articles were (a) lower prosthesis, (b) prosthetic leg, (c) prosthetic knee, (d) knee prostheses, and (e) active knee. To recognize appropriate articles and ensure that all the chosen articles satisfied the inclusion criteria as defined by the key search terms, the authors screened the title and abstracts of all computer-generated search results articles.  Figure 1 summarizes the search results, in which 838 potentially relevant titles and abstracts were identified in all the mentioned databases. Out of those, after reading through the titles and abstracts, 132 studies were identified as fulfilling the primary inclusion criteria. Upon further examination of each article based on the review criteria, three types of studies were excluded: ankle and foot prosthesis (29 studies), transtibial prosthesis (42 studies), and bipedal robot (7 studies).

**Figure 2:**
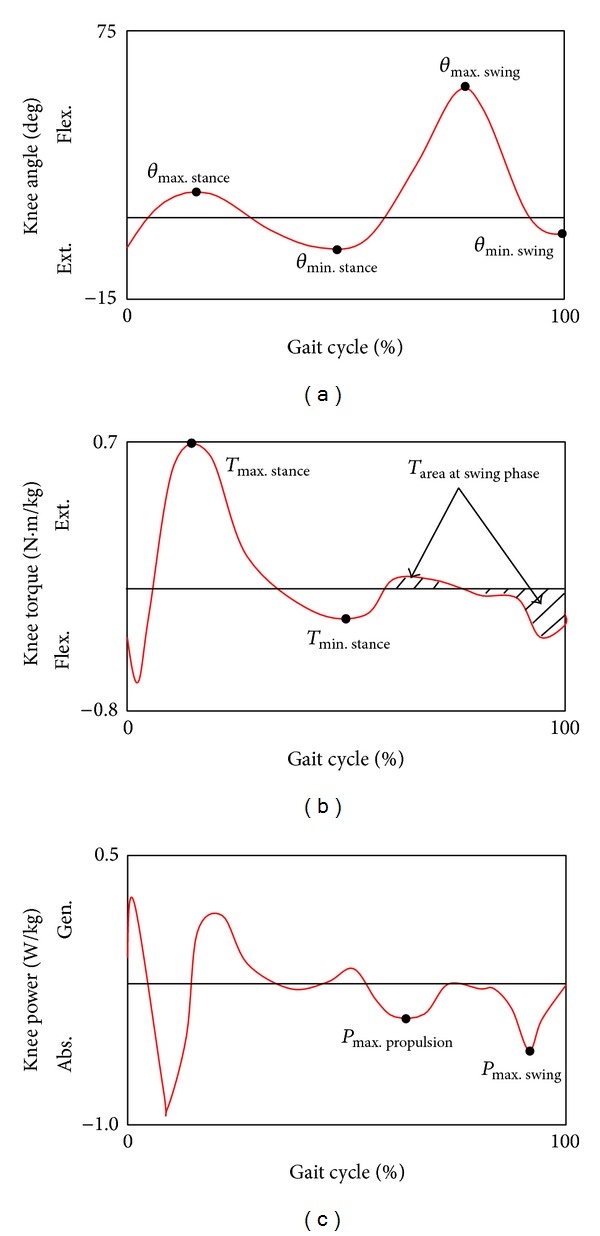
(a) Angle, (b) torque, and (c) power of the knee throughout the gait cycle and including various nine subparameters. Gait profiles were based on work of Segal et al., 2006 [26].

Only studies that reported clinical evaluation were included as they described the performance of the knee parameters during gait cycle. The performance parameters were later used in the efficacy assessment of the prosthetic knee devices by comparing them with the normal parameters. Other studies were excluded, as illustrated in Figure 2. Strategies that have been followed to quantitatively determine the walking performance were made. First, the intrinsic kinetic and kinematic parameters of the prosthetic knee, that is, movement angles (*θ*), moment or torque (*T*), and power (*P*), were identified [26] to represent a complete set of performance indicators as shown in Table 1. Those parameters were further divided into nine subparameters based on the full gait cycle profile as shown in Figure 2.

Four parameters were extracted from the knee angle profile: maximum stance angle (*θ*_max. stance_), minimum stance angle (*θ*_min. stance_), maximum swing angle (*θ*_max. swing_), and minimum swing angle (*θ*_min. swing_). These points that were chosen as the range of angle sensors were identified and selected based on the four angle parameters. To predict the required torque of the knee actuator, the following parameters were identified: maximum stance flexion (*T*_max. stance flex._), minimum stance extension (*T*_min. stance ext._), and summation of positive and negative torque during swing phase in terms of the area under the curve (*T*_Area_). To determine the functional performance of the prosthesis in terms of power, the full gait cycle power's profile was referred to the maximum power during propulsion (*P*_max. propulsion_) and maximum power during swing (*P*_max. swing_) selected as the comparable parameters. The power values provided an indication of the amount of generated and absorbed mechanical power during propulsion and swing phases, respectively [31]. Calculations of the average values for the subparameters (angle, torque, and power) were made by extracting those values from three specific published materials [6, 32, 33]. Figure 2 illustrates the nine parameters and the typical points of those parameters at different normal gait phases [6, 32, 33].

To objectively measure the functional performance, the nine subparameters were derived from the systematically retrieved studies and then compared to their respective points of normal gait profile curves as shown in Figure 2. The relative deviations from normal parameters were calculated using (1) to (3):

(1) $$\begin{array}{c} \theta =\left|\frac{\theta^{s}}{\theta^{n}}\right|, \end{array}$$

where *θ* is *θ*_max. stance_, *θ*_min. stance_, *θ*_max. swing_, or *θ*_min. swing_ while *θ* ^*s*^ and *θ* ^*n*^ refer to the knee angles obtained from each of the derived studies and normal knee angle, respectively. The same criterion was adopted to calculate the knee torque and power deviation percentage as shown in (2) and (3):

(2) $$\begin{array}{c} T=\left|\frac{T^{s}}{T^{n}}\right|, \end{array}$$

where *T* refers to either *T*_max. stance flex._ or *T*_min. stance ext._ points or *T*_Area_ at swing phase which refers to the area under the curve of the gait torque profile during the swing phase and *T* ^*s*^ and *T* ^*n*^ refer to knee torque in each of the derived studies and normal knee torque, respectively, and

(3) $$\begin{array}{c} P=\left|\frac{P^{s}}{P^{n}}\right|, \end{array}$$

where *P* is either *P*_max. propulsion_ or *P*_max. swing_ and *P* ^*s*^ and *P* ^*n*^ refer to knee power at each of the retrieved studies and normal knee power, respectively. Calculations based on (1) through (3) determined the value of the nine parameters as a percentage of the normal values for knee angle, torque, and power. The average values for the nine parameters were calculated individually from all obtained studies and presented in Table 1. For further details, see the Appendix.

A general weighted average equation to calculate the relative deviation of each knee parameter, *Q*, is shown in

(4) $$\begin{array}{c} Q=\frac{\sum_{j=1}^{Z}N_{j}Q_{j}}{\sum_{j=1}^{Z}N_{j}}, \end{array}$$

where *Q*_*j*_ is the value of each parameter *Q* in a particular study *j*, *Q* refers to each of the nine subparameters (*θ*_max. stance_, *θ*_min. stance_, *θ*_max. swing_ or *θ*_min. swing_, *T*_max. stance flex._ or *T*_min. stance ext._, *T*_Area_, *P*_max. propulsion_, or *P*_max. swing_), *N* is the number of the subjects participated in each study *j*, and *Z* is the number of studies.

A sample calculation of *θ*_max⁡. stance_ for one of the studies employing angle sensor (S1p) is illustrated as in (5):

(5) $$\begin{array}{c} \;\frac{18^{{}^{\circ}}\theta^{n}}{20^{{}^{\circ}}}=0.9\;\theta^{n}, \end{array}$$

where 18° is *θ*_max⁡. stance_ reported in the particular study [31] and 20° is the normal value of *θ*_max. stance_. As there were multiple studies that reported S1p, the average has been calculated as shown in (6) and the *Q*, in this case *θ*_max. stance_ value, of S1p was presented in Table 1:

(6) $$\begin{array}{c} Q=\frac{0.9\;\theta^{n}+0.35\;\theta^{n}+0.9\;\theta^{n}+0.9\;\theta^{n}+0.35\;\theta^{n}}{5}=0.68\;\theta^{n}. \end{array}$$

## 3. Results

As the current methodology will be useful in the development of active prosthetic knee devices, attention was given to the intrinsic thus controllable knee kinematic and kinetic parameters from different knee components such as sensors, actuators, and control algorithm. After exclusions, 53 studies were identified as being relevant to this research on microcontroller-based transfemoral prosthesis. Out of those, 24 studies reported the utilization of interactive extrinsic techniques and these were excluded. Those extrinsic techniques entailed that the system offered a level of communication between the user and the device, which may encompass afferent and efferent communication. This may include EMG signals from the muscles or other efferent systems that allow communication from the brain to be integrated in the control loop [2, 34]. Control of transfemoral amputee by measuring the EMG from the hamstring and quadriceps muscles has been examined and the results indicated appropriate knee motion during non-weight bearing activity [35]. Control of prosthetic knee based on EMG measurement was conducted for stair ascent with a powered transfemoral prosthesis, in which EMG measurements were performed to control the extension and flexion activity of muscles. Proportional knee control was implemented for unilateral prosthesis during stair ascent [17]. These studies were excluded because no gait trial performance was reported. After the initial stage, all the prior studies were disregarded and only seven studies were found to report both components: (i) development details and (ii) at least one amputee subject walking; thus, these were selected for further investigation. Other studies were excluded based on the reasons illustrated in Figure 2.

Table 2 summarizes the deviation in gait parameters reported from all seven studies, which were categorized by the three main parameters of the knee, namely, the angle, torque, and power, with respect to the types of sensors used, actuators employed, and the control method adopted. These draw direct relationships between each sensor, actuator, and control method utilized by each development study and the respective amputees' gait deviation. *Q* values closest to 1 indicate the least deviation from normal; thus components with values closest to one are perceived to be the most successful components in producing a close to normal gait.

Table 2 shows the normalized knee gait parameters of individual components, in which different sensors, actuators, and control techniques were listed and the *Q* values closest to 1 for different knee subparameters were highlighted, and asterisk values. Therefore, values closer to normal for every subparameter indicate the effect of technology in terms of sensors, actuators, and control techniques for future development of prosthetic knees. The nine subparameters of the kinematics and kinetics for the knee during the gait cycle were presented with respect to the technology that was utilized in each prosthetic knee device. The use of angle sensor potentiometer helped to increase the behavior accuracy of most of the knee parameters, as shown in the first row of Table 2.

The development of lower prosthesis that mimics natural knee movement is a challenge, especially when selecting an actuating mechanism that is capable of delivering stability and stiffness to the prosthesis.

## 4. Discussion

This study focused on the overall effect of each technology component, namely, sensors, actuators, and control technique that were incorporated in the various reported active prosthetic knee systems for transfemoral prosthesis. One might argue that it might be inaccurate or nonrepresentative to evaluate single components such as sensors, actuators, and controllers independently, as they all work as a whole system. In other words, the end gait performance of the system is a product of the whole synergy and it might not be accurate to claim that a better or worse performance is the singular effect of each component. Nevertheless, most components were reportedly used in multiple systems as presented in the studies retrieved. This allows one to deduce the potential performance of each component when it is integrated as part of any other system. On the other hand, many other microcontroller-based prosthetic knee systems have been developed that did not report any gait-related kinetics and kinematics performance, such as the Ossur Power Knee, Rheo Knee, and its combination with the Proprio Foot adaptive ankle system [36]. This had limited the ability of this study to compare the performance of the components from other reported developmental-based studies. Other developments may be either patented or not scientifically published and may not have been assessed by clinical trials. Nevertheless, since the results of this study were derived from a systematic approach where all available potential articles were extensively searched, conclusions from this study were deemed the best quantitative prediction that can be made. Technologies are evolving rapidly in this area; however, the assessment of the lower prosthesis will basically depend on the sensory system, power, and control.

This paper included 7 studies based on five technologies. The five technologies out of the seven retrieved studies reported utilization of different actuation mechanisms, namely, (1) series elastic actuators [6, 37, 38], (2) rotary motor driven ball screws [39, 40], (3) pneumatic actuated mechanisms [41], (4) linear step motor actuating four bar linkages [9], and (5) rotary motors driven three bar linkages [14]. These technologies proceeded to clinical trials [6, 37, 39, 41–44].

Agonist-antagonist active knee prosthesis [6, 38] contained different type of components configurations compared to the adaptive ones such as motors and springs. Series-elastic actuators were developed in agonist-antagonist configuration that adapt to the knee joint during movement. The technique of series elastic actuators was used in developing both ankle prosthesis and orthoses systems [34, 45]. In addition, FSR sensors located below the prosthetic foot were used to detect the gait instants. The advantages of the agonist-antagonist active knee are enhanced knee stability and adaptation to different ambulatory speeds [6, 17, 38]. The disadvantage is that some sensors are attached far from the knee axis such as force sensitive resistor insole [6, 38].

A linear step motor actuating four bar linkages was used to develop active knee prosthesis utilizing robotic control theories in the strategy of controlling the prosthesis. Using linear motor reduces the size and the weight of the system [9], while iterative learning control algorithm was used to mimic the prosthesis during stance and swing phase. Results shown in Table 2 indicated that the movement parameters of the knee joint using DC motors, with specific transmission mechanism to actuate the knee, improved the asterisk values subparameters. A computable control system is also an essential part in the development of active lower prostheses. Suitable control techniques enhanced most subparameters of the knee as revealed in the highlighted values in Table 2.

The Vanderbilt leg [39] was developed by incorporating the knee and ankle prosthesis in the whole lower limb system [13]. The architecture of the powered leg is considered as an embedded system. Two motors were utilized for both knee and ankle joint movement. Load cell was custom designed and incorporated between the user and the socket for purpose of intention derivation. Strain gauges were attached at the prosthetic foot to detect gait transitions. Although the powered leg was evaluated for walking, few trials were also conducted for other ADLs such as stair/ascent, stand/sit, and slope climbing [10] including one study that utilized the control of the knee during stair ascent by using EMG signals [14]. Their work is highly relevant as the knee system requires minimal power during normal walking as compared to the ankle. Thus, the power generated by the knees is less crucial during walking but is more relevant to be generated during stair ascent, stair descent, sit-to-stand, and climbing ramps.

While it is important to identify and evaluate the performance of each sensory, actuation and control element of a powered knee system, a more crucial element in designing the powered lower prosthesis is to build the powered knee joint that has the capability of performing functional tasks such as sit-to-stand and stair climbing. Researches nowadays are investigating better prosthetic devices to assist the amputees during sit-to-stand and stair climbing. The functional capability that requires modulation of the power output of the limb should be achieved with the knee joint to contain the motorized actuators, sensors, and the controller. The following sections discuss the components of various technologies utilized in the retrieved studies.

### 4.1. Sensors

A prosthetic knee system requires many input variables to perform optimally; thus, all utilized sensors were discussed in the current study. To select appropriate sensory systems for the knee devices, the control variables have to be determined. Adaptive-control of prosthetic knee [42] was reported to monitor two types of variables, namely, force/moment and flexion parameter. Microprocessor acquires information from the sensors to vary the resistance of the knee movement during gait. Strain gauges were located at appropriate positions close to the knee axis to identify a suitable correlation of the knee joint movement. Angle sensors were attached at the knee axis to measure the knee extension/flexion during walking. The sensors were used to duplicate the normal gait cycle for the adaptive-control prosthetic knee by varying the impedance. Active artificial knee joint [37] provides the tracking position of the knee joint during the gait cycle. Measuring the knee flexion and identifying the appropriate knee position during gait cycle can assist tracking of the knee position reference trajectory. Angle rotary sensor was attached to be able to perform that task. Agonist-antagonist active knee prosthesis [6, 38] used knee angle sensor, hall effect sensor, and FSR. Different impedance was identified by the controller during the stance and swing phases. All the described technologies related to the mentioned researches employed different sensory systems integrated with the knee devices based on the controlled parameters.

Angle sensor potentiometer (S1p) has reported relatively acceptable values according to the nine subparameters of the knee (Table 1). The potentiometer is used as an angle sensor to measure knee angles at every state; it delivers satisfactory results that directly affect the performance of the knee, especially with regards to maximum stance and maximum swing knee angles. One of the advantages of the potentiometer is that it is low in cost and easily integrated with other components as its output signal can be easily processed. One promising integration method was achieved by incorporating the potentiometer with an accelerometer or gyroscope in order to measure the accurate inclination of a knee when climbing or ascending stairs or navigating sloped walking terrains [34]. Moment or torque sensor (S3) has shown good *Q* values at most of the torque and propulsion power. However, its kinematic performance was only acceptable for maximum stance and maximum swing angles. On the other hand, the technology that uses position sensor or hall sensor (S4) has shown reliable results for the knee angle parameters and only some torque and power measurements, that is, minimum knee stance torque and maximum knee propulsion power.

Both angle sensor and moment or torque sensors play a prominent role in developing an active knee. In order to record heel strike and toe-off to provide information about walking phases, FSR is used, located directly below the prosthetic foot. Furthermore, it is low in price, relatively thin, small, and produces analog-based signal [46]. The FSR sensor (S2f) is a pressure sensor that is used to detect the walking phase of the prosthetic knee, according to the S2f results for minimum stance, maximum stance, and minimum swing, essential in enhancing knee movement. In terms of loading, FSR delivers sufficient information about different walking phases; but as the FSR is located below the prosthetic foot itself, replacement of the foot would require technical adjustment, which may be quite impractical amongst normal prosthetists or amputee users themselves. Ideally, all sensors should be included at a location near to the knee axis in order to adequately control the prosthetic knee. In one study [36], axial force sensors (two anterior and two posterior strain gauges) measured the component of force applied to the knee prosthesis from the ground in the direction of the knee's longitudinal axis. The same strain gauge sensors were used to measure the torque [42]. While most configurations are quite successful so far, it still lacks the interaction between user and device, especially in detecting intention. A lot of studies presented EMG as the intention or interaction sensor, but discussion on these techniques is beyond the scope of this paper. It may be worth considering mechanical-based sensor to be placed inside the socket closest to the user itself instead of EMG to detect ground pressure and user initiated movements.

### 4.2. Actuators

The natural knee produces an internal flexor moment, due to contraction of the hamstrings, which prevents hyperextension at the end of the swing phase. As the knee starts to flex, concentric contraction of the hamstrings, as well as the release of energy stored in the ligaments of the extended knee, results in short-lived power generation [47]. For amputees who have lost their lower limb, utilizing an appropriate actuator system such as a motor could facilitate walking during stance and swing phases, particularly by providing energy at the beginning of the swing phase. Actuators used in damping strategies [42] can be categorized as hydraulic, pneumatic, and magnetorheological (MR). The motorized actuation technique was used in prosthetic knee technology to deliver positive power to assist the amputee in performing some ADLs such as generating sufficient power during stair ascent/descent, and stand/sit, as well as slope climbing. Motors are connected to gear box and lead screw assembly to generate the required moment and knee power.

All actuator types are listed with different *Q* values (Table 2). The motor connected to ball screw mechanism (A1s) presented the most decent values during the measurements of knee angle, torque, and power. Pneumatic cylinder controlled by servovalve (A2v) also shows an acceptable deviation from the normal for angle, torque, and power. According to the results, scientific studies using A1s indicated better results compared to A2v. The use of magnetorheological actuator (A3) was only reported in one study [36], with limited information on the clinical trial, especially in terms of torque and power. However, in terms of the angle, it has acceptable *Q* values.

### 4.3. Control Method

Control strategy is considered as the most critical part in a power prosthetic knee; control strategy is not needed in nonpowered prostheses. Impedance control algorithm was the most commonly used control strategy, in which the torque generated is adapted to the produced knee angle. This mode of control ensures that the knee joint generates torque that is suitable for each gait phase [6, 41, 43]. Another type of control algorithm was tracking control, whereby the joint was made to follow or track a predefined trajectory [9]. This tracking method controls angle and velocity of the knee joint during stance and swing phase. As shown in Table 2, various control techniques have been used in prosthetic devices. The finite-state-based impedance control approach (C3) demonstrates the most acceptable results for all knee parameters. The modulated torque enabled the user to interact with the prosthesis according to the phase of walking that he or she is engaged in [6]. Finite state control (C1) also delivered acceptable *Q* values, especially the swing phase knee angles, but only 1 study [6] reportedly used the control method; thus, the results were deemed less reliable than C3.

**Figure 3:**
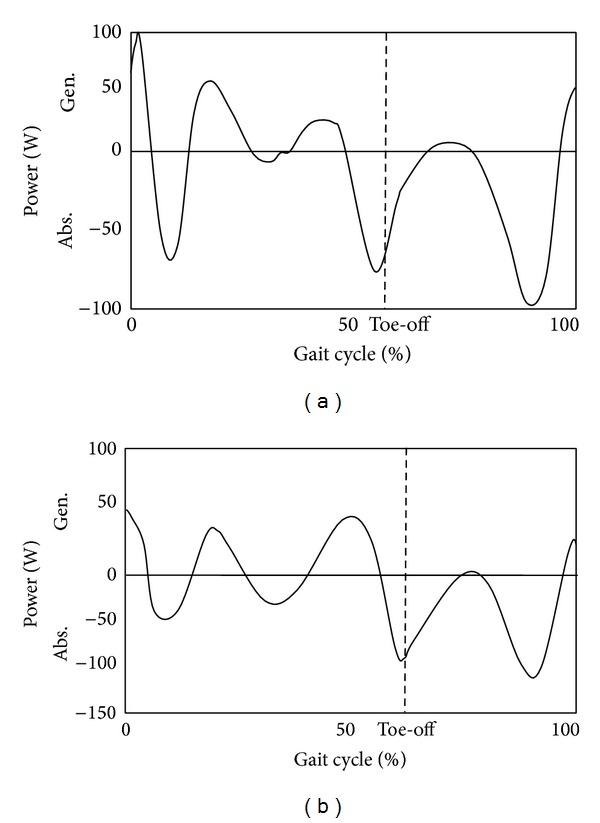
Knee power during normal walking, (a) and (b) [6, 38], respectively.

**Figure 4:**
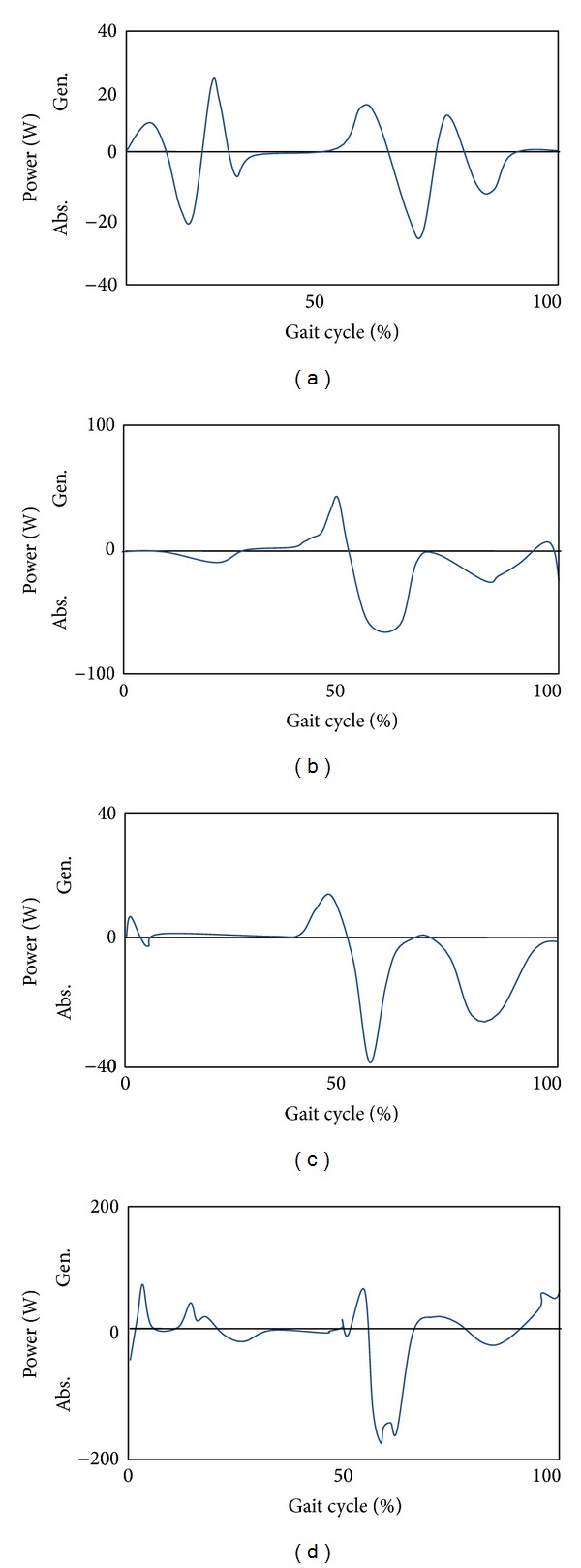
Knee power of different prosthetic knee systems, (a)–(d) adapted from four studies [6, 39, 40, 43].

For further enhancement of the prosthetic knee movement, all possible sensors that detect different knee variables should be in the prosthetic knee structure containment. The problem concerns not only the selection of a specific sensor, actuator, or control method but also the integration and synchronization between various components. By reviewing the knee power results for both normal and prosthetic knees, as shown in Figures 3 and 4, respectively, the authors were able to identify subtle differences in both the normal and prosthetic systems; due to the adaptation in muscle activities. This is most apparent at the beginning of the swing phase, as can be seen in the prosthetic knee system detailed in Figure 4.

Another conclusion that can be made in terms of the knee power as one of the important parameters which affect the knee performance is illustrated in Figure 3. The first peak at the start of the swing phase is higher than the second one in the same phase (Figure 3). This can be explained by the fact that the normal gait involved muscles that naturally provide the normal flexion/extension process to control the rate of power output production throughout the gait cycle. However, the knee power shown in Figure 3 was produced by active knee systems and does not have the advantage of antagonist muscle extension to “brake” the knee angular acceleration. In other words, the active knee systems do not contain eccentric contraction of the rectus femoris in their artificial structure. Thus, the rate of knee flexion can only be controlled by power absorption at the artificial knee. The same occurs for the second peak at swing phase where the amount of power absorption is less than that for the previous peak as there are no hamstrings that contract to prevent the knee from hyperextending. Acknowledging these issues enables realistic development and optimization of control algorithm in future works.

### 4.4. Weight Considerations

One of the critical considerations in constructing lower limb prosthesis is the weight of the prosthesis. Nowadays, researchers try to optimize the weight during the development of the prosthetic knee. In addition, accompanying devices and weight reduction, strength and functionality are usually the primary goals in prosthetic fabrication. Metal is mainly used for rotating components whereas aluminum as an alternative to steel is used conservatively for smaller components. Titanium is more expensive; however, due to its biocompatibility, it is considered for some aspects of prosthetic devices [48].

Lower limb prostheses need to support the body weight during the stance phase and must prevent the knee from sudden joint flexion [42]. Lighter prosthetic knee may also provide more comfort to the user when he or she walks on sloped terrains or up and down stairs. The overall weight of the active prosthesis can be reduced by minimizing the overall volume of the selected actuators [41]. Knee prosthesis should not exceed the size and weight of the missing limb; thus, the weight of the transfemoral prosthesis plays an essential role in its development.  Table 3 classified the corresponding weight of the prosthesis of the current devices.

Amputees can produce energy for their prosthetic leg by utilizing active energy harvesting device in the system. Such techniques may decrease the device's dependency on a battery and therefore may reduce the overall weight of the prosthesis. Incorporating new kinds of light material such as thermoplastics within structural components is a breakthrough in prosthesis fabrication [48]. Rigid polypropylene was used for structural support. Polyethylene, being more flexible, was used in the prosthetic interface with the residual limb, providing a more comfortable and adjustable socket. Copolymers, which can be heated and reshaped after initial fabrication to accommodate, for example, the changes in the residual limb, are also of increasing importance.

### 4.5. Prosthetic Foot Type

Prosthetic foot is considered one of the prosthetic knee components which may have affected the biomechanical outcomes at the knee joint [49]. Thus, a brief idea about the prosthetic foot systems that were incorporated in the lower prosthesis systems is presented herein (Table 4). Prosthetic foot devices affect the selection of the suitable control scheme of the lower prosthesis at different locomotion such as ground-level walking, sitting/standing mode, and stair ascent/descent [50]. Overall, most systems used conventional passive prosthetic foot except Sup et al. [39], who utilized a custom sensorized foot.

### 4.6. Power Supply

The sensors and actuators that are incorporated in the knee system need electrical power source for their operation and the power supply is another crucial component in microcontroller-based prosthetic knee devices. The power sources that are mentioned in the available studies are listed in Table 5. Modern trends in active knee systems utilize new power sources that are capable of delivering up to 5.2 V. An example of this can be seen in the use of piezoelectric material as an active power generating material. This technology might decrease the weight of the battery while saving some power and can also be utilized as an emergency power source [51, 52].

In order to achieve a more realistic prosthetic knee system for transfemoral amputees, additional sensors might be integrated in the knee system to recognize the intention of the amputee's movement. Combining gyroscopes with an accelerometer to recognize the orientation and acceleration of the knee joint in *x*- and *y*-axes is a potential solution. Moreover, during stair climbing or slope walking activities, the sensors would be reliable in delivering instantaneous signals to the controller to adapt to the prosthetic knee movement. From a biomechatronics point of view, the integration of sensors and electric actuators such as electric motors is easier in terms of communication and interaction due to the same domain of electrical operating signals. This reduces the need for additional hydraulic or pneumatic sources.

The control of lower limb prosthesis presents another significant challenge, especially during transition between phases. One example was illustrated in robotic ankle device that was capable of adapting its mechanical movement according to different walking conditions [46, 53]. The main challenge in adopting similar technology in a prosthetic knee device, that is, to be able to identify precisely the transition between different speed and gait modes, for example from normal walking to stair ascent/descent [30] the control scheme and actuation system have to response to the variation of human factors in the transfemoral prosthesis users. The control strategy should be linked with the body by using interface between body and prosthesis, and that may enhance control of the whole prosthesis. Possible techniques for that interface include surface electromyographic interface and implantable peripheral nervous system interface [54].

By using robotic control algorithm, in which the controller supervises the trajectory of the joint position, it is possible to improve the ability of prosthesis to mirror the performance of a healthy leg profile [8]. Iterative learning control (ILC) is a relatively new technique that utilizes angle and angular velocity to control prosthetic knee joint [9]. ILC offer a control framework that is capable of ensuring stable and coordinated interaction with the user and his or her external environment. Although these advances add to the complexity of the smart transfemoral prosthesis design in general, they offer more accuracy, greater simplicity, and adaptability for the end amputee users' benefit.

## 5. Conclusion

In this paper, the efficacy of adaptive dissipative or powered-based prosthetic knee systems was reviewed based on the contribution of the sensor, actuators, and control technique towards the production of a normal gait profile. From the 7 retrieved studies, the knee's potentiometer was identified as the most commonly used sensor while its performance was comparably reliable as the FSR, moment sensor, and hall position sensors in measuring knee parameters. The DC electric motor connected to a ball screw mechanism was identified as the most used actuator with most reliable performance for the development of the powered or motorized prosthetic knee. These evaluations were performed by comparing the gait cycle behavior of the prosthetic knee systems with the normal gait cycle in terms of the nine subparameters of the knee. Minimum deviation from normal gait parameters was also derived from the application of the impedance control method, which in turn was suggested as the best technique for controlling prosthetic knee during different gait phases.

Finally, the biomechatronics design approach was based on integrating the prominent sensors, actuators, and control techniques that were identified within this review; smart active prosthetic knee systems might be developed to strategically integrate all the identified components. Additional sensors could be embedded within the prosthetic knee system to enhance the interaction between the user and the environment. An alternative technique can be made by incorporating sensory system embedded inside the socket wall to receive direct information from the residual limb and further enhance the interaction between the user and the device as well as user intention. Furthermore, the knee controller could monitor the knee movement and intention based on contraction and retraction of the residual muscles, as well as the resultant force experienced at the stump from the body and the ground reaction forces. Similarly, the user can produce some of the electrical power required by using active elements such as piezoelectric material to produce the amount of electrical power needed to operate the electronic devices [55]. Ultimately, development of separate powered knee and ankle devices that are capable of working either together or independently is deemed more beneficial for the general transfemoral and transtibial amputee populations.

Based on the current state of literature, it can be safely concluded that most research-based studies have not clinically validated performance with the real intended users. Therefore, identification of the best technology can only be limitedly made. This highlights the need for more clinical trials to be performed for the newly developed prosthetic knee systems to provide validated assessment of the prosthesis, especially in mimicking the biological gait across range of modes such as stair ascent and descent and ramp or uneven terrain walking.

## Figures and Tables

**Table 1:** Gait biomechanics parameters identified as performance indicator.

Major parameters	Subparameters	Calculated normal values
Knee angle	Maximum stance angle (*θ*_max. stance_)	20° [6, 32, 33]
	Minimum stance angle (*θ*_min. stance_)	2° [6, 32, 33]
	Maximum swing angle (*θ*_max. swing_)	64.5° [6, 32, 33]
	Minimum swing angle (*θ*_min. swing_)	0.5° [6, 32, 33]

Knee torque	Maximum stance flexion torque (*T*_max. stance flex._)	4 N*·*m [6, 32]
	Minimum stance extension torque (*T*_min. stance ext._)	20 N*·*m [6, 32]
	Area under the swing phase torque curve (*T*_Area_)	117 N*·*m [6, 32]

Knee power	Maximum propulsion power (*P*_max. propulsion_)	73.08 W [6, 33]
	Maximum swing power (*P*_max. swing_)	97.4 W [6, 33]

**Table 2:** Normalized knee gait parameters, *Q*, of individual components in active prosthetic systems.

	Knee angle				Knee torque			Knee power	
	*θ*_max. stance_	*θ*_min. stance_	*θ*_max. swing_	*θ*_min. swing_	*T*_max. stance flex._	*T*_min. stance ext._	*T*_Area_	*P*_max. propulsion_	*P*_max. swing_
Sensors									
S1p [37, 39, 41–43]	0.68*θ* ^*n*^	2.38*θ* ^*n*^	1.03*θ* ^*n*^	3.2*θ* ^*n*^	7.69*T* ^*n*^	1.14*T* ^*n*^	0.93*T* ^n^*	1.41*P* ^*n*^	0.2*P* ^*n*^
S2f [6]	0.5*θ* ^*n*^	0.5*θ* ^n^*	0.98*θ* ^n^*	0.95*θ* ^n^*	5*T* ^*n*^	0.75*T* ^*n*^	0.2*T* ^*n*^	0.44*P* ^*n*^	0.12*P* ^*n*^
S2s [42]	0.35*θ* ^*n*^	NV	0.9*θ* ^*n*^	2*θ* ^*n*^	NV	NV	NV	NV	NV
S3 [39, 41, 43]	0.72*θ* ^n^*	1.83*θ* ^*n*^	1.08*θ* ^*n*^	4.67*θ* ^*n*^	4.33*T* ^n^*	1.18*T* ^*n*^	1.07*T* ^n^*	1.06*P* ^n^*	0.18*P* ^*n*^
S4 [6]	0.5*θ* ^*n*^	0.5*θ* ^n^*	1.03*θ* ^*n*^	0.95*θ* ^n^*	11.38*T* ^*n*^	0.89*T* ^n^*	0.31*T* ^*n*^	1.27*P* ^*n*^	0.18*P* ^*n*^

Actuators									
A1e [6]	0.5*θ* ^*n*^	0.5*θ* ^n^*	0.95*θ* ^*n*^	0.95*θ* ^n^*	5*T* ^*n*^	0.75*T* ^n^*	0.2*T* ^*n*^	0.44*P* ^*n*^	0.12*P* ^*n*^
A1b [39]	0.9*θ* ^n^*	1.5*θ* ^n^*	1.13*θ* ^*n*^	2*θ* ^*n*^	8.75*T* ^*n*^	1.8*T* ^*n*^	1.95*T* ^*n*^	NV	NV
A1s [39, 41]	0.9*θ* ^n^*	3.75*θ* ^*n*^	1.02*θ* ^n^*	4*θ* ^*n*^	9.75*T* ^*n*^	0.69*T* ^*n*^	0.83*T* ^n^*	1.63*P* ^*n*^	0.23*P* ^*n*^
A2s [44]	NV	NV	1.15*θ* ^*n*^	NV	NV	NV	NV	NV	NV
A2v [41]	0.35*θ* ^*n*^	0.5*θ* ^n^*	0.98*θ* ^n^*	6*θ* ^*n*^	2.5*T* ^n^*	1.4*T* ^*n*^	0.12*T* ^*n*^	1.1*P* ^n^*	0.135*P* ^*n*^
A3 [42]	0.35*θ* ^*n*^	NV	0.9*θ* ^*n*^	2*θ* ^*n*^	NV	NV	NV	NV	NV

Control method									
C1 [6]	0.5*θ* ^*n*^	0.5*θ* ^*n*^	0.95*θ* ^n^*	0.95*θ* ^n^*	5*T* ^*n*^	0.75*T* ^*n*^	0.1*T* ^*n*^	0.44*P* ^*n*^	0.12*P* ^*n*^
C2 [37]	0.9*θ* ^n^*	4*θ* ^*n*^	0.93*θ* ^*n*^	2*θ* ^*n*^	NV	NV	NV	NV	NV
C3 [39, 41, 43]	0.72*θ* ^*n*^	1.83*θ* ^n^*	1.06*θ* ^*n*^	4.67*θ* ^*n*^	7.33*T* ^*n*^	0.92*T* ^n^*	0.59*T* ^*n*^	1.41*P* ^n^*	0.2*P* ^*n*^
C4 [44]	NV	NV	1.15*θ* ^*n*^	NV	1.75*T* ^n^*	0.35*T* ^*n*^	1.15*T* ^n^*	NV	NV
C5 [42]	0.35*θ* ^*n*^	NV	0.9*θ* ^*n*^	2*θ* ^*n*^	NV	NV	NV	NV	NV

S1p: angle sensor, S2f: FSR (force sensitive resistive), S2s: strain gauge, S3: moment sensor or torque sensor, S4: position sensor, and NV: no value reported from study. A1e: electric motor with series elastic springs, A1b: brushless motor, A1s: motor connected to ball screw, A2s: pneumatic cylinder controlled by stepper motor, A2v: pneumatic cylinder controlled by servovalve, and A3: magnetorheological actuator. C1: finite state control, C2: proportional, integral, and derivative (PID), C3: finite-state-based impedance control approach, C4: iterative learning control (ILC), and C5: adaptive control scheme. Values with asterisk indicate the closest to normal values for each of the nine gait parameters of the knee, that is, *Q* closest to 1. Based on all nine values for each sensor, actuator, and controller, it can be deduced that most sensors (S1p, S2f, S3, and S4) are all acceptable sensors, motor connected to ball screw (A1s) is the most appropriate actuator, and finite-state-based impedance control approach (C3) would most likely perform as the best controller when combined to other sensor and actuating components.

**Table 3:** Weight of the prosthetic knee systems.

Author	Description of the system, weight of the volunteer, and walking speed	Weight of the prosthesis/system
Martinez-Villalpando and Herr, 2009 [6]	Volunteer mass = 97 kg and walking speed = 0.81 m/s.	≈3 kg, including the weight of lost segments, titanium, aluminum, and polyamide materials.

Kapti and Yucenur, 2006 [37]	Titanium, aluminum, and polyamide materials were used. Volunteer mass and walking speed were not mentioned.	6.9 kg.

Sup et al., 2007 [41]	A tethered transfemoral prosthesis with pyramid connectors. Volunteer mass = 75 kg and walking speed = 0.675 m/s.	2.65 kg.

Sup et al., 2008 [43]	Volunteer mass = 85 kg and walking speed = slow, normal, and fast (2.2, 2.8, and 3.4 km/hr), respectively.	3.8 kg.

Fite et al., 2007 [40]	Volunteer mass = 85 kg and walking speed = 0.675 m/s.	3.05 kg, including the foot and pyramid connectors of the tethered transfemoral prosthesis.

Torrealba et al., 2010 [56]	Volunteer mass = not mentioned and walking speed = self-selected speed.	2.0591 kg.

Sup et al., 2009 [39]	Volunteer mass = 80 kg and walking speed = 5.1 km/h.	4.2 kg, the whole prosthetic knee system including sensors, actuators, and electronics.

Gong et al., 2010 [44]	Volunteer mass = 62 kg, walking speed = slow, normal, and fast (0.66, 0.74, and 1.21 m/s), respectively.	Not mentioned.

Geng et al., 2010 [9]	Volunteer mass = 62 kg, walking speed = slow, normal, and fast of average values (1, 1.19, and 1.48 m/s), respectively.	Not mentioned.

Wu et al., 2011 [17]	Volunteer mass = 83 kg, stair ascent.	3.5 kg, including electronics and batteries.

**Table 4:** Prosthetic foot devices in the prosthetic knee systems.

Author	Prosthetic foot type
Martinez-Villalpando and Herr, 2009 [6]	Conventional passive-elastic ankle-foot prosthesis (Flex-Foot LP-VariFlex from Össur [Reykjavik, Iceland]).
Kapti and Yucenur, 2006 [37]	Prosthetic foot (no specific type mentioned).
Gong et al., 2010 [44]	Prosthetic foot (no specific type mentioned).
Sup et al., 2009 [39]	Custom sensorized prosthetic foot.
Wu et al., 2011 [17]	Commercial low-profile prosthetic foot (Lo Rider, Otto Bock, Germany).
Sup et al., 2008 [43]	Custom sensorized prosthetic foot.
Fite et al., 2007 [40]	Low profile prosthetic foot (Otto Bock, Lo Rider).
Torrealba et al., 2010 [56]	Prosthetic foot (no specific type mentioned).

**Table 5:** Power sources in the prosthetic knee systems.

Author	Power source for various prosthetic knee systems	Weight
Martinez-Villalpando and Herr, 2009 [6]	The system was powered by a 6-cell Lithium polymer battery (22.2 V nominal).	0.15 kg.

Gong et al., 2010 [44]	Rechargeable lithium ion battery.	Not mentioned.

Sup et al., 2009 [39]	A lithium polymer battery with 29.6 V nominal rating and 4000 mA*·*h capacity.	0.825 kg.

Fite et al., 2007 [40]	A high-power Li-ion battery from A123 Systems Inc., (model ANR26650MI) for onboard power where the nominal capacity of a single battery is 2.3 Ah and 3.3 V.	0.07 kg.

Wu et al., 2011 [17]	Four 11.1-V, 2000-mAh lithium polymer batteries.	0.122 kg each.

**Table 6:** Detailed average calculations for different knee parameters versus various sensors.

Sensor (S)	Knee angle				Knee torque			Knee power	
	*θ*_max. stance_	*θ*_min. stance_	*θ*_max. swing_	*θ*_min. swing_	*T*_max. stance flex._	*T*_min. stance ext._	*T*_Area_	*P*_max. propulsion_	*P*_max⁡. swing_
S1p			(0.93*θ* ^*n*^) [31],						
	(0.9*θ* ^*n*^) [31], (0.35*θ* ^*n*^) [34], (0.9*θ* ^*n*^) [35], (0.9*θ* ^*n*^) [42], (0.35*θ* ^*n*^) [37]	(4*θ* ^*n*^) [31], (0.5*θ* ^*n*^) [34], (3.5*θ* ^*n*^) [35], (1.5*θ* ^*n*^) [42]	(0.98*θ* ^*n*^) [34], (1.13*θ* ^*n*^) [35], (1.15*θ* ^*n*^) [41], (1.13*θ* ^*n*^) [42], (*θ* ^*n*^) [36], (0.9*θ* ^*n*^) [37]	(2*θ* ^*n*^) [31], (4*θ* ^*n*^) [34], (6*θ* ^*n*^) [35], (2*θ* ^*n*^) [42], (2*θ* ^*n*^) [37]	(2.5*T* ^*n*^) [34], (1.75*T* ^*n*^) [35], (8.75*T* ^*n*^) [42], (17.75*T* ^*n*^) [36]	(1.4*T* ^*n*^) [34], (0.35*T* ^*n*^) [35], (1.8*T* ^*n*^) [42], (1.02*T* ^*n*^) [36]	(0.12*T* ^*n*^) [34], (1.15*T* ^*n*^) [35], (1.95*T* ^*n*^) [42], (0.51*T* ^*n*^) [36]	(1.1*P* ^*n*^) [34], (1.02*P* ^*n*^) [35], (2.1*P* ^*n*^) [36]	(0.13*P* ^*n*^) [34], (0.23*P* ^*n*^) [35], (0.23*P* ^*n*^) [36]
Average	0.68*θ* ^*n*^	2.38*θ* ^*n*^	1.03*θ* ^*n*^	3.2*θ* ^*n*^	7.69*T* ^*n*^	1.14*T* ^*n*^	0.93*T* ^*n*^	1.41*P* ^*n*^	0.2*P* ^*n*^

S2f	(0.5*θ* ^*n*^) [5]	(0.5*θ* ^*n*^) [5]	(0.95*θ* ^*n*^) [5], (*θ* ^*n*^) [30]	(0.95*θ* ^*n*^) [5]	(5*T* ^*n*^) [5]	(0.75*T* ^*n*^) [5]	(0.2*T* ^*n*^) [5]	(0.44*P* ^*n*^) [5]	(0.12*P* ^*n*^) [5]
Average	0.5*θ* ^*n*^	0.5*θ* ^*n*^	0.98*θ* ^*n*^	0.95*θ* ^*n*^	5*T* ^*n*^	0.75*T* ^*n*^	0.2*T* ^*n*^	0.44*P* ^*n*^	0.12*P* ^*n*^

S2s	(0.35*θ* ^*n*^) [37]		(0.9*θ* ^*n*^) [37]	(2*θ* ^*n*^) [37]	NV	NV	NV	NV	NV
S3	(0.35*θ* ^*n*^) [34], (0.9*θ* ^*n*^) [35], (0.9*θ* ^*n*^) [42]	(0.5*θ* ^*n*^) [34], (3.5*θ* ^*n*^) [35], (1.5*θ* ^*n*^) [42]	(0.98*θ* ^*n*^) [34], (1.13*θ* ^*n*^) [35], (1.135*θ* ^*n*^) [42]	(6*θ* ^*n*^) [34], (6*θ* ^*n*^) [35], (2*θ* ^*n*^) [42]	(2.5*T* ^*n*^) [34], (1.75*T* ^*n*^) [35], (8.75*T* ^*n*^) [42]	(1.4*T* ^*n*^) [34], (0.35*T* ^*n*^) [35], (1.8*T* ^*n*^) [42]	(0.12*T* ^*n*^) [34], (1.15*T* ^*n*^) [35], (1.95*T* ^*n*^) [42]	(1.1*P* ^*n*^) [34], (1.02*P* ^*n*^) [35]	(0.135*P* ^*n*^) [34], (0.23*P* ^*n*^) [35]
Average	0.72*θ* ^*n*^	1.83*θ* ^*n*^	1.08*θ* ^*n*^	4.67*θ* ^*n*^	4.33*T* ^*n*^	1.18*T* ^*n*^	1.07*T* ^*n*^	1.06*P* ^*n*^	0.18*P* ^*n*^

S4	(0.5*θ* ^*n*^) [5],	(0.5*θ* ^*n*^) [5],	(0.95*θ* ^*n*^) [5], (*θ* ^*n*^) [36], (1.15*θ* ^*n*^) [41]	(0.95*θ* ^*n*^) [5]	(5*T* ^*n*^) [5], (17.75*T* ^*n*^) [36]	(0.75*T* ^*n*^) [5], (1.02*T* ^*n*^) [36]	(0.1*T* ^*n*^) [5], (0.51*T* ^*n*^) [36]	(0.44*P* ^*n*^) [5], (2.1*P* ^*n*^) [36]	(0.12*P* ^*n*^) [5], (0.23*P* ^*n*^) [36]
Average	0.5*θ* ^*n*^	0.5*θ* ^*n*^	1.03*θ* ^*n*^	0.95*θ* ^*n*^	11.38*T* ^*n*^	0.89*T* ^*n*^	0.31*T* ^*n*^	1.27*P* ^*n*^	0.18*P* ^*n*^

S1p: angle sensor, S2f: FSR (force sensitive resistive), S2s: strain gauge, S3: moment sensor or torque sensor, S4: position sensor, and NV: no value reported from study.

**Table 7:** Detailed average calculations for different knee parameters versus actuators.

Actuator (A)	Knee angle				Knee torque			Knee power	
	*θ*_max. stance_	*θ*_min. stance_	*θ*_max. swing_	*θ*_min. swing_	*T*_max. stance flex._	*T*_min. stance ext._	*T*_Area_	*P*_max. propulsion_	*P*_max. swing_
A1e	(0.5*θ* ^*n*^) [5]	(0.5*θ* ^*n*^) [5]	(0.95*θ* ^*n*^) [5]	(0.95*θ* ^*n*^) [5]	(5*T* ^*n*^) [5]	(0.75*T* ^*n*^) [5]	(0.2*T* ^*n*^) [5]	(0.44*P* ^*n*^) [5]	(0.12*P* ^*n*^) [5]
A1b	(0.9*θ* ^*n*^) [42]	(1.5*θ* ^*n*^) [42]	(1.13*θ* ^*n*^) [42]	(2*θ* ^*n*^) [42]	(8.75*T* ^*n*^) [42]	(1.8*T* ^*n*^) [42]	(1.95*T* ^*n*^) [42]	NV	NV
			(0.93*θ* ^*n*^) [31]						
A1s	(0.9*θ* ^*n*^) [31], (0.9*θ* ^*n*^) [35],	(4*θ* ^*n*^) [31] (3.5*θ* ^*n*^) [35]	(1.13*θ* ^*n*^) [35] (*θ* ^*n*^) [36]	(2*θ* ^*n*^) [31], (6*θ* ^*n*^) [35]	(1.75*T* ^*n*^) [35], (17.75*T* ^*n*^) [36]	(0.35*T* ^*n*^) [35] (1.02*T* ^*n*^) [36]	(1.15*T* ^*n*^) [35] (0.51*T* ^*n*^) [36]	(1.15*P* ^*n*^) [35] (2.1*P* ^*n*^) [36]	(0.23*P* ^*n*^) [35], (0.23*P* ^*n*^) [36]
Average	0.9*θ* ^*n*^	3.75*θ* ^*n*^	1.02*θ* ^*n*^	4*θ* ^*n*^	9.75*T* ^*n*^	0.69*T* ^*n*^	0.83*T* ^*n*^	1.63*P* ^*n*^	0.23*P* ^*n*^

A2s	NV	NV	(1.15*θ* ^*n*^) [41]	NV	NV	NV	NV	NV	NV
A2v	(0.35*θ* ^*n*^) [34]	(0.5*θ* ^*n*^) [34]	(0.98*θ* ^*n*^) [34]	(6*θ* ^*n*^) [34]	(2.5*T* ^*n*^) [34]	(1.4*T* ^*n*^) [34]	(0.12*T* ^*n*^) [34]	(1.1*P* ^*n*^) [34]	(0.135*P* ^*n*^) [34]
A3	(0.35*θ* ^*n*^) [37]	NV	(0.9*θ* ^*n*^) [37]	(2*θ* ^*n*^) [37]	NV	NV	NV	NV	NV

A1e: electric motor with series elastic springs, A1b: brushless motor, A1s: motor connected to ball screw, A2s: pneumatic cylinder controlled by stepper motor, A2v: pneumatic cylinder controlled by servovalve, and A3: magnetorheological actuator.

**Table 8:** Detailed average calculations for different knee parameters versus control methods.

Control technique (C)	Knee angle				Knee torque			Knee power	
	*θ*_max. stance_	*θ*_min. stance_	*θ*_max. swing_	*θ*_min. swing_	*T*_max. stance flex._	*T*_min. stance ext._	*T*_Area_	*P*_max. propulsion_	*P*_max. swing_
C1	(0.5*θ* ^*n*^) [5]	(0.5*θ* ^*n*^) [5]	(0.95*θ* ^*n*^) [5]	(0.95*θ* ^*n*^) [5]	(5*T* ^*n*^) [5]	(0.75*T* ^*n*^) [5]	(0.1*T* ^*n*^) [5]	(0.44*P* ^*n*^) [5]	(0.12*P* ^*n*^) [5]
C2	(0.9*θ* ^*n*^) [31]	(4*θ* ^*n*^) [31]	(0.93*θ* ^*n*^) [31]	(2*θ* ^*n*^) [31]	NV	NV	NV	NV	NV
C3	(0.35*θ* ^*n*^) [34], (0.9*θ* ^*n*^) [35], (0.9*θ* ^*n*^) [42]	(0.5*θ* ^*n*^) [34], (3.5*θ* ^*n*^) [35], (1.5*θ* ^*n*^) [42]	(0.98*θ* ^*n*^) [34], (1.13*θ* ^*n*^) [35], (1.135*θ* ^*n*^) [42], (*θ* ^*n*^) [36]	(6*θ* ^*n*^) [34], (6*θ* ^*n*^) [35], (2*θ* ^*n*^) [42]	(2.5*T* ^*n*^) [34], (1.75*T* ^*n*^) [35], (17.75*T* ^*n*^) [36]	(1.4*T* ^*n*^) [34], (0.35*T* ^*n*^) [35], (1.02*T* ^*n*^) [36]	(0.12*T* ^*n*^) [34], (1.15*T* ^*n*^) [35], (0.51*T* ^*n*^) [36]	(1.1*P* ^*n*^) [34], (1.02*P* ^*n*^) [35], (2.1*P* ^*n*^) [36]	(0.135*P* ^*n*^) [34], (0.23*P* ^*n*^) [35], (0.23*P* ^*n*^) [36]
Average	0.72*θ* ^*n*^	1.83*θ* ^*n*^	1.06*θ* ^*n*^	4.67*θ* ^*n*^	7.33*T* ^*n*^	0.92*T* ^*n*^	0.59*T* ^*n*^	1.41*P* ^*n*^	0.2*P* ^*n*^

C4	NV	NV	(1.15*θ* ^*n*^) [41]	NV	(1.75*T* ^*n*^) [41]	(0.35*T* ^*n*^) [41]	(1.15*T* ^*n*^) [41]	NV	NV
C5	(0.35*θ* ^*n*^) [37]	NV	(0.9*θ* ^*n*^) [37]	(2*θ* ^*n*^) [37]	NV	NV	NV	NV	NV

C1: finite-state control, C2: proportional, integral, and derivative (PID), C3: finite-state-based impedance control approach, C4: iterative learning control (ILC), and C5: adaptive control scheme.

## Appendix

See Tables 6, 7, and 8.
